# Deep Learning Based Pile-Up Correction Algorithm for Spectrometric Data Under High-Count-Rate Measurements

**DOI:** 10.3390/s25051464

**Published:** 2025-02-27

**Authors:** Yiwei Huang, Xiaoying Zheng, Yongxin Zhu, Tom Trigano, Dima Bykhovsky, Zikang Chen

**Affiliations:** 1 Shanghai Advanced Research Institute, Chinese Academy of Sciences, Shanghai 201210, China; huangyw@sari.ac.cn (Y.H.); chenzk@sari.ac.cn (Z.C.); 2 University of Chinese Academy of Sciences, Beijing 101408, China; 3 Electrical and Electronics Engineering Department, Shamoon College of Engineering, Be’er Sheva 8410802, Israel

**Keywords:** high count rate, pulse pile-up, deep learning, nuclear spectroscopy

## Abstract

Gamma-ray spectroscopy is essential in nuclear science, enabling the identification of radioactive materials through energy spectrum analysis. However, high count rates lead to pile-up effects, resulting in spectral distortions that hinder accurate isotope identification and activity estimation. This phenomenon highlights the need for automated and precise approaches to pile-up correction. We propose a novel deep learning (DL) framework plugging count rate information of pile-up signals with a 2D attention U-Net for energy spectrum recovery. The input to the model is an Energy–Duration matrix constructed from preprocessed pulse signals. Temporal and spatial features are jointly extracted, with count rate information embedded to enhance robustness under high count rate conditions. Training data were generated using an open-source simulator based on a public gamma spectrum database. The model’s performance was evaluated using Kullback–Leibler (KL) divergence, Mean Squared Error (MSE) Energy Resolution (ER), and Full Width at Half Maximum (FWHM). Results indicate that the proposed framework effectively predicts accurate spectra, minimizing errors even under severe pile-up effects. This work provides a robust framework for addressing pile-up effects in gamma-ray spectroscopy, presenting a practical solution for automated, high-accuracy spectrum estimation. The integration of temporal and spatial learning techniques offers promising prospects for advancing high-activity nuclear analysis applications.

## 1. Introduction

**Figure 1:**
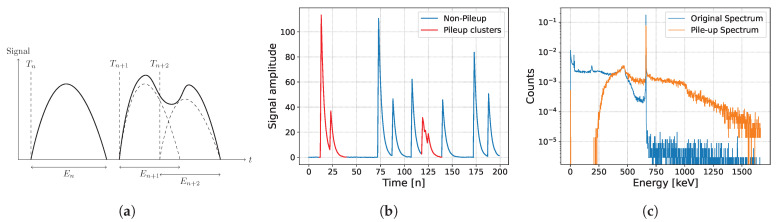
An example of signal pile-up phenomenon and its effect on analyzing spectrum. At a count rate of 0.05, that is $0.05\times 10^{7}$ cps, the duty cycle is given by 0.82 with a theoretical pile-up probability of 0.558 (numerical details are introduced in Section 4). (**a**) Illustration of electrical pulses and their stacking phenomenon. (**b**) Raw pile-up signal and pile-up clusters (red). (**c**) The true spectrum of Cs137 (blue) and the distorted spectrum (orange) were obtained by directly calculating the pile-up clusters.

Gamma-ray spectroscopy is an important tool in the field of nuclear science, enabling the identification and characterization of radioactive materials by analyzing the energy spectra of emitted gamma rays. This technique is widely used in various applications, including nuclear security, environmental monitoring, and geological exploration. Analyzing gamma-ray spectra, particularly at high count rates, is often complicated by the “pile-up” effect, as illustrated in Figure 1. The overlap of electrical pulses, particularly present at high activities, results in spectral distortions and inaccuracies in the estimation of activity [1]. Various correction strategies have been developed to address the pile-up issue. The most common approach is pile-up rejection, which detects pile-up events and discards them to retain individual pulses only [2]. Although effective, this method inevitably increases the duration of experiments and can lead to a loss of valuable data. Some methods attempt to compensate for rejected pulses in the time domain by correcting the start point of the pile-up pulse, as referenced in [3,4]. Other techniques include pulse clipping [5] and template fitting [6]. These methods are typically most effective in systems with low pile-up probabilities and minimal pulse-shape dictionaries. Other research has considered the output signal as a linear combination of known shapes, relating the pile-up effect to a sparse regression problem [7].

Rather than recognize individual pulses in the time domain, recent efforts seek to analyze pulse collections; in [8], the authors formulated a non-linear inversion problem and developed a non-parametric pile-up correction method. In [9], the authors reformulated the pile-up problem as a decompounding problem, constructing a kernel-based estimator to model pulse pile-up. Similarly, ref. [10] used the characteristic function of the energy spectrum to infer the distribution of gamma photon energies from indirect measurements.

In addition to pile-up problems, traditional methods for isotope identification rely on the expertise of nuclear scientists who manually discriminate between background and source photopeaks, adjust calibrations, and account for shielding effects [11]. The subjective nature of this analysis and the increasing demand for automated, real-time solutions have driven the exploration of machine learning techniques to address these challenges. In particular, deep neural networks (DNNs) have emerged as a powerful approach to automated gamma-ray spectroscopy. These algorithms can learn complex patterns from the data, mimicking the intuitive decision-making process of experienced professionals. They have shown promising results in the identification of gamma-ray spectra under various conditions, including large calibration drifts and unknown background radiation fields [12]. The application of DNNs to spectroscopy has been extensive, with uses in peak fitting, isotope identification, and activity estimation [13]. Jeon et al. [14] developed a convolutional neural network (CNN) that attempts to separate and estimate the true pulse height of pile-up events from high-count-rate radiation. A DL-based technique for detecting and classifying pulses under high count rates was proposed in [15]. In [16], a CNN-based automated isotope identification method was proposed. In [17], another DL-based architecture was proposed for activity estimation. A pulse height analysis technique based on deep learning using U-net was introduced in [18].

This study proposes a DL-based method for processing pulse pile-up clusters, enabling accurate spectrum estimation for different sources at very high count rates. Our method focuses on the pile-up correction algorithm. Specifically, we construct the Energy–Duration histogram of the pile-ups signals observed over a certain time period as the model input and employ a hybrid estimation model combining attention U-Net and known signal activity information, embedding activity information into the neural network to predict the corrected energy spectrum. Our training data are generated from an open-source simulator [19,20]. Our experiments show that under very high activity conditions of $2.95\times 10^{6}$ count per second (cps), and of pile-up probability 0.996, the proposed hybrid DL estimation model is capable of recovering severe pile-up spectrum of various sources and achieving accurate prediction results.

Our contribution can be summarized as follows:

1. Spectrum Estimation Model for High Count Rates: A novel model is proposed for pile-up correction and spectrum estimation for high-count-rate scenarios. It achieves accurate predictions even in heavily distorted conditions, addressing the challenges in high-activity gamma source analysis;
2. Innovative Input Design with Energy–Duration Matrix: The Energy–Duration matrix, constructed via zero-crossing segmentation, is introduced as the input for 2D-UNet. This design effectively represents the mandatory information for pile-up correction;
3. Hybrid Model Combining Temporal and Spatial Features: This work integrates activity information and 2D-UNet architectures to extract temporal dependencies and spatial features from pulse signals. The embedding of count rate information enhances the robustness and accuracy of spectrum recovery under high count rate conditions.

The paper is organized as follows. We introduce the signal formulation and problem model in Section 2. The proposed DL algorithm is outlined in Section 3 and the experiments are carried out in Section 4, including the simulation details, and the model’s performance evaluations are detailed in Section 5.

## 2. Problem Formulation

In this section, we introduce the problems that this paper aims to solve, including the theory of signal composition, the explanation of the pile-up effect, the generation method of training and test data, and the construction method of the Energy–Duration matrix.

### 2.1. Pile-Up Description and Correction Theory

In gamma-ray spectroscopy, “pile-up” refers to the phenomenon where multiple events, such as pulse signals, overlap within a short time window due to a high count rate, causing the signals to be hard to separate. This phenomenon is commonly observed when the detector’s response time is limited, or its signal processing capacity is insufficient. Pile-up affects the energy spectrum’s accuracy and can lead to spectrum distortion or loss of signals.

Assume that the detector records multiple events $x_{i}\left(t\right)$ within a given time window. The detector’s overall response y(t) will be the sum of the signals stemming from all the events. This can be expressed as:

(1) $$y\left(t\right)=\sum_{i=1}^{N}x_{i}\left(t-t_{i}\right),$$

where $x_{i}\left(t\right)$ represents the signal from the *i*-th event, $t_{i}$ is the time stamp of that event, and *N* is the number of events that occur within the current time window. To understand the impact of pile-up on energy measurements, let $E_{i}$ denote the energy of the *i*-th event. In the case of pile-up, the total energy $E_{total}$ will be the sum of the energies of *N* overlapping events, which can be written as:

(2) $$E_{total}=\sum_{i=1}^{N}E_{i}.$$

Let $E_{\mathrm{true}}=\left\{E_{1},E_{2},\ldots ,E_{N}\right\}$, if the true energies in set $E_{\mathrm{true}}$ belongs to particular bins $b(E_{i})$, the pile-up energy $E_{total}$ may instead fall into an incorrect bin $b(E_{total})$, leading to a distortion in the histogram:

(3) $$H\left(E\right)=\sum_{i=1}^{N}\delta \left(b\left(E_{i}\right)\right),$$

where δ is the Dirac delta function that places each signal’s energy in its corresponding bin. In the case of pile-up, the distribution of $E_{total}$ across the bins will be incorrect, leading to errors in the energy statistics and, ultimately, in the interpretation of the spectrum, such as the generation of artificial peaks or the suppression of true peaks.

Our goal is to correct the $E_{true}$ from $E_{distorted}$, where $E_{distorted}=\left\{E_{total_{1}},E_{total_{2}},\ldots E_{total_{n}}\right\}$. To address this issue, similar to [21], we regard this as a nonlinear mapping problem:

(4) $$E_{true}=f\left(E_{distorted}\right),$$

where *f* is non-linear because the energy distribution of pile-up events is influenced by various factors, such as the detector’s response time, the time interval between overlapping events, and the energy DL model to solve the pile-up correction problem.

### 2.2. Signal Formula

In order to create our dataset, we rely on synthetic signals, simulated in the time domain as follows. The detector output can be modeled as a linear combination of pulse shapes. A signal consisting of *N* events can be modeled as follows:

(5) $$X\left(n\right)≜\sum_{k\geq 1}^{N}E_{k}\phi_{k}\left(n-T_{k}\right)+\varepsilon \left(n\right),$$

where $E_{k}$ represents each photon’s where energy which is drawn randomly from an energy distribution based on known spectrum data *H*, $T_{k}$ is the sample path of a homogeneous Poisson process with constant known intensity λ, ε(n) denotes the additive Gaussian noise, and pulse shape $\phi_{k}$, is randomly chosen from a dictionary *A*. The signal construction rules are described as follows

**Figure 2:**
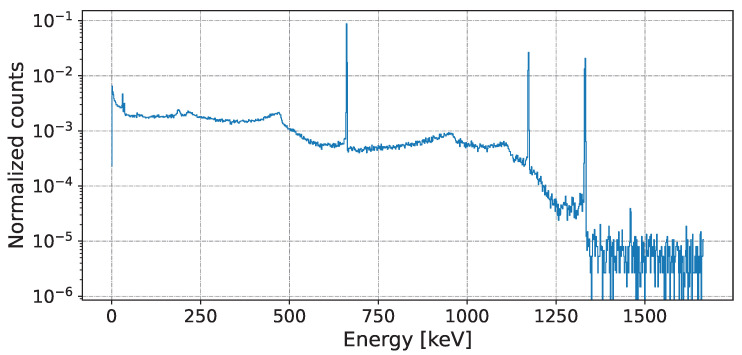
An example of a 1:1 mixture of Cs137 and Co60.

1. Shape dictionary: One of the common shape models is the double exponential [1] which can be modeled as:
  (6) $$\begin{array}{cc} h(t) & =a\left(\exp\left(-\frac{t}{\tau_{2}}\right)-\exp\left(-\frac{t}{\tau_{1}}\right)\right), \end{array}$$
  where *a* is the normalization factor and $\tau_{1}\ll \tau_{2}$ are characteristic decay times. For practical purposes, any pulse shape is assumed to have a finite time length T. A key characteristic of the pulse shape is the peak time, $t_{r}$, also known as the rise time, which is complementary to the fall time $t_{f}=T-t_{p}$. For instance, the rise time for the double exponential model is given by:
  (7) $$t_{r}=\frac{\tau_{1}\tau_{2}}{\tau_{2}-\tau_{1}}\ln\frac{\tau_{2}}{\tau_{1}},$$
  where $\tau_{1}\ll \tau_{2}$ are the decay times. In practice, a single precise parameter set cannot perfectly fit the pulse shape due to the physical inhomogeneities, temperature fluctuation, and other factors, leading us to generate our parameter set randomly from a Gaussian distribution with reasonable expectations and variances [22].
2. Arrival times: as mentioned above, the arrival of gamma particles is modeled using a Poisson counting process with a constant intensity of λ. The inter-arrival times follow an exponential distribution with expectation 1/λ.
3. Source: The spectrum of the signal can be either single source or multi-sources, the interactions among a mixture of multiple elements can be formed as:
  (8) $$P\left(E=e\right)=\sum_{i}^{n}\frac{w_{i}}{\sum_{i}w_{i}}P_{e_{i}}\left(E=e\right),$$
  where $P_{e_{i}}$ represents the probability that $e_{i}$ emits energy *e*, *E* is the signal source’s spectrum, and $w_{i}$ is the proportion of source $e_{i}$. Thus, the spectrum of the mixture sources can be modeled as the following:
  (9) $$H_{mix}=\sum_{i}^{n}\frac{w_{i}}{\sum_{i}w_{i}}E_{i}.$$
  By doing so, the probability density function of the mixture histogram can be derived.
4. Energy–Probability: The common representation of the probability of arrival particles’ energies per energy bin $H_{i}$ (typically measured by keV) is by a histogram that represents the energies of a series of signal events. This energy histogram can be easily modeled by a discrete random distribution where the probability of energy is proportional to $b_{i}$:
  (10) $$\mathrm{Pr}\left(H=H_{i}\right)=\frac{b_{i}}{\sum_{i}^{B}b_{i}},$$
  where $b_{i}$ is the number of events with $i_{th}$ energy, *B* represents the bin index, and the pairs of values for $H_{i}$ and $b_{i}$ of a single source or mixed source can be determined by the accept–reject method [23]. An example of a mixed spectrum is shown in Figure 2.

Pile-up events occur when multiple pulses appear within a single trace. To assess the probability of pile-up, the duty cycle (DC) value is used as a convenient parameter. The DC is calculated by multiplying the arrival count rate by the pulse shape length:

(11) DC=λT.

Based on the probability relation,

(12) $$\mathrm{Pr}\left(N\left(t\right)=n\right)=\frac{{\left(\lambda t\right)}^{n}}{n!}\exp-\left(\lambda t\right),$$

the resulting pile-up probability P is given by:

(13) P=Pr(N(T)>0)=1−Pr(N(T)=0)=1−exp(−DC).

Well-developed standard methods can be used to generate sequences that fit a particular exponential distribution [23].

In this study, we consider DC values above 0.1 to have a high pile-up probability.

### 2.3. Dataset Generation

We used different sources and their mixtures and different activities to generate pile-up signals based on a Gamma spectroscopy database and an open-source simulator. The sources are chosen from [Co60, Cs137, Ba133, Co57, Eu152, Na22, Ac225, Am241, Ba131, Cr51, Er169, Hg197m, I124, I125, I131, K40, La138, Lu176,Pb210, Ra226, Re186, Re188, Sm153]. The raw spectrum from the database is 8192 bins, and the maximum energy is 1666 keV; to simplify the representation and calculations, we processed the 8192 bins of data into 1024 bins by merging adjacent bins together, and we described the spectra using bin index rather than the energy values. The detailed parameters are listed in Table 1.

### 2.4. Pulse Signal Segmentation

The time signals are first processed by detecting zero-crossing points, which are used to segment the signals into pulse clusters. Zero-crossing points are identified as the points where the signal transitions from positive to negative (or vice versa). The signal is then divided into segments $\left\{s_{1},s_{2},\ldots ,s_{N}\right\}$, where each segment $s_{i}$ is bounded by two consecutive zero-crossing points.

The energy $E_{i}$ of each segment $s_{i}$ is computed as the sum of the absolute values of the signal within that segment:

$$E_{i}=\sum_{n\in s_{i}}\left|X\left(n\right)\right|$$

The duration $D_{i}$ of each segment is simply the length of the segment in terms of time:

$$D_{i}=\mathrm{length}\left(s_{i}\right)$$

Thus, each segment is characterized by its energy $E_{i}$ and duration $D_{i}$ that contain either a pile-up of individual pulses or a single individual pulse.

### 2.5. Energy–Duration Matrix Construction

Based on [21], the minimal information required for pile-up correction is the couple durations/energies, and there exists a non-linear relation linking the 2D histogram of the pile-ups to the 2D histogram of the individual pulses. Since DNN architectures can estimate non-linear relations efficiently, it seems logical to use this 2D representation. To construct the input matrix for the neural network, the energy and duration values of all pulse clusters are accumulated into an Energy–Duration matrix *M*. The matrix is indexed by energy and duration, with each element M(e,d) representing the count of pulse segments that have energy *e* and duration *d*.

Each segment $s_{i}$ is assigned a position $\left(e_{i},d_{i}\right)$ in the matrix corresponding to its energy $E_{i}$ and duration $D_{i}$, and the matrix is updated through a period time L of raw signal as follows:

$$\mathrm{for}\;\mathrm{each}\;i:\;M\left(e_{i},d_{i}\right)\leftarrow M\left(e_{i},d_{i}\right)+1$$

This results in a sparse matrix *M*, where the value at position (e,d) represents the frequency of pulse segments with energy *e* and duration *d*.

Overall, our goal can be summarized as follows: recover the true energy spectrum *H* from a short period of distorted pulse signals L by processing the Energy–Duration matrix *M* along with known count rate λ using a DL model. The optimization objective is to ensure that the output energy spectrum $\hat{H}$ predicted by the model closely matches *H* by minimizing the discrepancy between the predicted and actual spectra. We define the pile-up spectrum recovery problem as:

(14) $$\begin{array}{c} \min_{\theta }L\left(D(M(e,d);\lambda ,\theta ),H\right), \end{array}$$

where ${\hat{H}}_{i}=D\left(M\left(e,d\right);\lambda ,\theta \right)$ is the predicted energy spectrum at the *i*-th energy bin by model D, *H* is the true energy spectrum, L(·) represents the loss function, typically Mean Squared Error (MSE) measuring discrepancy between the predicted and true spectra, and we aim to find the optimal θ parameters of the model by optimizing the function.

## 3. Method

**Figure 3:**
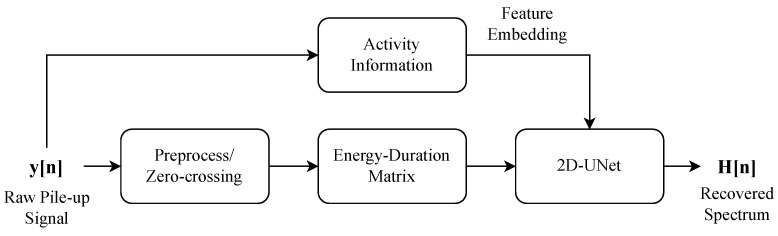
Workflow of our proposed method.

In this section, we introduce the workflow of the proposed novel approach, as shown in Figure 3, to recover the true energy spectrum from distorted pulse signals using a DL model that integrates the Energy–Duration matrix with real-time count rate information directly embedded into a 2D U-Net architecture. The overall methodology can be broken down into the following key steps:

1. Pulse Signal Preprocessing: The raw pulse signals are first processed to generate an Energy–Duration matrix, which is achieved by segmenting the signal based on zero-crossing points. The resulting Energy–Duration matrix contains the spatial characteristics of the signal and serves as a feature representation for the 2D U-Net model.
2. Embedding Count Rate Information into 2D U-Net: The true count rate information is directly embedded into the 2D U-Net model, representing the temporal feature, intuitively giving the model prior information about the intensity of signal pile-up. This allows the 2D U-Net to process both spatial and temporal features simultaneously.
3. Energy Spectrum Recovery: The 2D U-Net, now augmented with the Energy–Duration matrix and the count rate information, processes these inputs to generate the predicted energy spectrum. The output of the network is compared with the true energy spectrum during training, and the model is optimized to minimize the reconstruction error using Mean Squared Error (MSE).

By directly embedding count rate information into the bottleneck, as well as utilizing the pixel-level feature extract ability of 2D U-Net to process the fine-grained Energy–Duration matrix data, our approach streamlines the process of energy spectrum recovery, improving robustness and accuracy under high-count-rate conditions.

### 3.1. Count Rate Embedding Module

**Figure 4:**
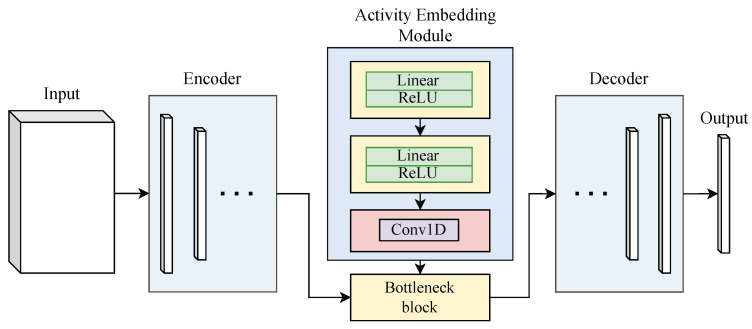
Schematic diagram of the neural network for embedding count rate features. The encoding and decoding of data are simply represented by white cubes.

We directly embed the true count rate information into the 2D U-Net architecture to enhance its capability to recover the true energy spectrum from distorted pulse signals, as shown in Figure 4. The count rate embedding module is implemented as a fully connected neural network followed by a convolutional layer, which transforms the scalar count rate value into a high-dimensional feature map that is compatible with the 2D U-Net bottleneck. The structure of the embedding module is as follows: A fully connected linear layer that maps the scalar input λ to a 16-dimensional space, enabling the model to learn an intermediate representation of the count rate. A ReLU activation function is applied after the first linear transformation, introducing non-linearity to facilitate complex feature learning. A second linear layer projects the 16-dimensional representation into a higher-dimensional space corresponding to the energy dimension of the input data, effectively encoding the count rate information. A 1D convolutional layer that processes the count rate feature map, producing an output with a size consistent with the duration of the signal, thus enabling spatial processing of the count rate features.

The processed count rate feature from the embedding module is integrated into the 2D U-Net bottleneck via tensor addition. Specifically, after the count rate embedding module generates a high-dimensional feature map matching the dimensions of the bottleneck tensor, the two tensors are added element-wise. This operation ensures that the count rate information is directly infused into the 2D U-Net’s intermediate representation, enabling the model to jointly consider both temporal dynamics (captured by the Energy–Duration matrix) and signal intensity (represented by the count rate).

By integrating the count rate information at the bottleneck, the model can jointly process the temporal dynamics (through the Energy–Duration matrix) and the signal intensity (via the count rate), leading to improved performance in estimating the true energy spectrum, particularly under high-count-rate conditions.

### 3.2. 2D-UNet

#### 3.2.1. Attention U-Net Model Architecture

**Figure 5:**
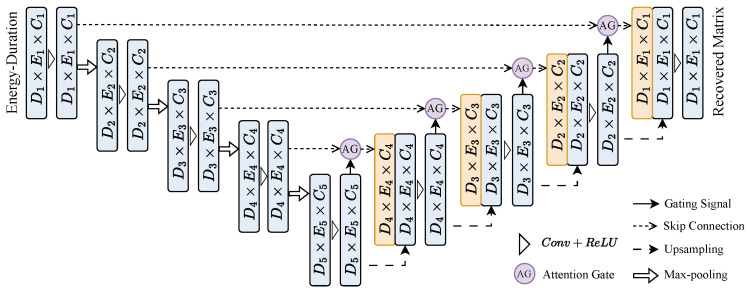
Architecture of the Attention U-Net model used for Energy–Duration matrix recovery. The model processes the input Energy–Duration matrix (size of $D_{1}\times E_{1}\times C_{1}$) through an encoder–decoder structure with skip connections and attention gates (AGs). The encoder extracts hierarchical features through convolution and max-pooling layers, reducing spatial dimensions (D,E) while increasing channel depth (*C*). The decoder progressively upsamples the feature maps, integrating encoder features via skip connections and attention mechanisms to selectively focus on relevant regions. The decoder output is the recovered matrix with the same spatial dimensions as the input.

The proposed model employs an Attention U-Net architecture [24], which consists of an encoder, a decoder, and attention gates applied to the skip connections between the encoder and decoder sub-networks, as shown in Figure 5. The attention mechanism in this architecture allows the network to focus on the most relevant parts of the input signal, improving its ability to learn meaningful features while discarding irrelevant information. This results in more accurate energy spectrum recovery, especially for high-dimensional data.

The encoder–decoder architecture is similar to that of the original U-Net [25] but with the key modification of incorporating attention gates. These gates allow the decoder to focus on specific regions of the feature maps received from the encoder, thus promoting the learning of multi-scale information and improving feature propagation.

Let $X\in R^{B\times E\times D\times C}$ represent the input matrix to the model, where *B* is the batch size, *E* is the energy size, *D* is the duration, and *C* is the number of channels. For each input instance $x\in R^{E\times D}$, the goal of the Attention U-Net model is to predict the energy spectrum vector $\hat{y}\in R^{L\times 1}$, where $\hat{y}$ is a vector of length L representing the corrected energy spectrum. The network is trained end-to-end to minimize the reconstruction error between the predicted and true energy spectra.

#### 3.2.2. Encoder Architecture

The encoder is responsible for extracting hierarchical feature representations from the input matrix X. It consists of several convolutional blocks, each followed by a ReLU activation and a max-pooling operation to downsample the feature maps. The feature maps are progressively downsampled in spatial resolution, but their depth (number of channels) is increased at each layer to capture more abstract features.

Let $f_{\mathrm{input}}=X$ be the input to the first convolutional layer of the encoder. At each subsequent layer, the output $f_{i}$ is computed as:

$$f_{i}=\mathrm{Conv}\left(\mathrm{ReLU}\left(\mathrm{Conv}\left(f_{i-1}\right)\right)\right)$$

where Conv(·) denotes a convolution operation and ReLU(·) is the rectified linear unit activation. The encoder reduces the spatial dimensions of the input matrix while increasing the feature depth, allowing it to learn increasingly complex patterns in the data.

#### 3.2.3. Decoder Architecture

The decoder reconstructs the output energy spectrum from the features extracted by the encoder. It consists of several transposed convolutional layers (also known as up-sampling or deconvolution layers), which progressively increase the spatial resolution of the feature maps. At each layer of the decoder, skip connections from the corresponding encoder layers are incorporated to retain high-resolution features.

The decoder’s input is the feature map from the last layer of the encoder, denoted as $f_{\mathrm{encoded}}$. The decoder progressively upsamples this feature map to recover the input’s spatial dimensions. The feature map at layer *i* of the decoder, denoted $f_{\mathrm{dec}}^{i}$, is computed as

$$f_{\mathrm{dec}}^{i}=\mathrm{Upsample}\left(\mathrm{Conv}\left(f_{\mathrm{dec}}^{i-1}\right)\right)$$

where Upsample(·) denotes an up-sampling operation, and Conv(·) is a convolution operation applied to the upsampled feature map.

#### 3.2.4. Attention Gates

In our case, the Energy–Duration matrix we constructed is typically sparse, in which the peak feature is fine-grained and spatial-related. The AG implementation enables the model to filter out irrelevant background regions and focus on the target sum-up peaks.

The key feature of the Attention U-Net architecture is the use of attention gates that control the flow of information through the skip connections. These gates allow the model to focus on important spatial regions by dynamically weighting the importance of the features received from the encoder.

The attention map $A_{i}$ is computed dynamically for each layer based on the feature maps passed through the attention gate. At each skip connection between the encoder and decoder, the feature map $f_{\mathrm{encoded}}^{i}$ is processed by an attention gate, which is defined as:

$$A_{i}=\sigma \left(f_{\mathrm{skip}}\cdot W_{\mathrm{skip}}+f_{\mathrm{encoded}}\cdot W_{\mathrm{enc}}\right),$$

where σ(·) is the sigmoid activation function, $W_{\mathrm{skip}}$ and $W_{\mathrm{enc}}$ are learnable weight matrices, and $f_{\mathrm{skip}}$ is the feature map from the decoder and $f_{\mathrm{encoded}}$ is the feature map from the encoder at layer *i*. In practice, the weight matrices $W_{\mathrm{skip}}$ and $W_{\mathrm{enc}}$ are initialized randomly and updated during training using backpropagation.

The output of AGs is the element-wise multiplication of input feature maps and attention maps:

$$f_{\mathrm{att}}^{i}=A_{i}⊙f_{\mathrm{skip}},$$

where ⊙ denotes element-wise multiplication. This operation selectively focuses on the important features while suppressing irrelevant ones.

Applying the attention gates allows the network to adjust its focus on different regions of the input, based on the spatial relevance of the features, which improves the recovery of important energy regions.

#### 3.2.5. Final Output Layer

The output of Attention U-Net’s decoder is a reconstructed de-pile-up energy spectrum feature. The final output $\hat{y}\in R^{L\times 1}$, is obtained by firstly applying a 1D convolutional layer to the output of the decoder which refines the feature representations, and followed by an adaptive average pooling operation for extracting features from a sparse matrix. Finally, the pooled features are flattened and passed through a multi-layer perceptron (MLP) to map the output to a vector of dimensions identical to the target energy spectrum

$${\hat{y}}_{hidden}=\mathrm{Conv}1D\left(f_{\mathrm{dec}}^{N}\right),$$

$$\hat{y}=\mathrm{MLP}\left(\mathrm{AvgPool}2d\left({\hat{y}}_{hidden}\right)\right)$$

where $f_{\mathrm{dec}}^{N}$ is the final feature map from the decoder, AvgPool2d is the adaptive average 2d pooling, Conv1D(·) is a 1D convolution operation, and ${\hat{y}}_{hidden}$ is the output spectrum’s hidden state. The output $\hat{y}$ represents the predicted energy spectrum, which is compared with the true spectrum during training.

### 3.3. Loss Function and Optimization

The optimization objective is to minimize the Mean Squared Error (MSE) between the predicted energy spectrum $\hat{y}$ and the true energy spectrum *y*:

(15) $$L\left(\hat{y},y\right)=\frac{1}{N}\sum_{i=1}^{N}{\left({\hat{y}}_{i}-y_{i}\right)}^{2},$$

where *N* is the number of energy bins, ${\hat{y}}_{i}$ and $y_{i}$ are the predicted and true energy values at the *i*-th energy bin.

## 4. Experiments

In this section, we describe the experimental setup, including the training and inference processes, and the datasets used for training the 2D U-Net model with count rate embedding.

### 4.1. Training Parameters

The total size of the dataset used for the training is 1800, we split the dataset into training (80%) and validation sets (20%), 1440 samples, and 360 samples, respectively. The training set consists of signals with activities in the range of [0.05, 0.3], with a step size of 0.01, while the dataset used for the test contains activities in the range of [0.05, 0.3] with a step size of 0.005, excluding the activities used for training. This setup ensures that the model’s generalization ability is evaluated on unseen activity levels.

The model is trained for 200 epochs using the Adam optimizer with an initial learning rate of $1\times 10^{-4}$. The batch size is set to 16, and training is performed using two GPUs in parallel. The learning rate is dynamically adjusted: if the validation loss does not improve, the learning rate is halved every 10 epochs. The training process is monitored to ensure convergence and prevent overfitting. Table 2 lists the detailed training parameters.

### 4.2. Hardware and Software Setup

All experiments were implemented using the PyTorch framework (https://pytorch.org/). Training was performed on two NVIDIA A30 GPUs in parallel. The model training and inference were carried out on a distributed system to speed up computation. The training environment included the use of mixed precision for efficient memory usage.

### 4.3. Evaluation Metrics

The performance of the 2D U-Net model was evaluated using four metrics: the Kullback–Leibler (KL) divergence, the Mean Squared Error (MSE), the Energy Resolution (ER), and the Full Width at Half Maximum (FWHM). These metrics were used to assess the accuracy of the predicted energy spectra on test datasets.

Kullback–Leibler (KL) divergence: The KL divergence is used to measure the difference between two distributions. We use the symmetric KL divergence (${KL}_{s}$) to evaluate the probability distribution distance between the predicted spectra and the reference spectrum:

(16) $$D_{KL}\left(P||Q\right)=\sum_{i=1}^{B}P\left(i\right)\cdot \log\left(\frac{P(i)}{Q(i)}\right),$$

and

(17) $${KL}_{s}=\left[\frac{1}{2}\sum_{i=1}^{B}\left(\mathrm{KL}\left(P_{i},∥Q_{i}\right)+\mathrm{KL}\left(Q_{i}∥P_{i}\right)\right)\right].$$

Mean Squared Error (MSE):

(18) $$\mathrm{MSE}=\frac{1}{B}\sum_{i=1}^{B}{\left(S_{i}-{\hat{S}}_{i}\right)}^{2}$$

Energy Resolution (ER): We define ER to evaluate the accuracy of the model in estimating the position of key peaks in the energy spectrum, which is defined as the relative difference between the estimated peak energy and the true peak energy:

$$\mathrm{ER}=\frac{|E_{\mathrm{estimated}}-E_{\mathrm{true}}|}{E_{\mathrm{true}}}$$

Additionally, we compare the performance of our method against two conventional methods:

1. Method 1: A baseline method that does not correct for pulse pile-up.
2. Method 2: A fast correction algorithm, as described in [21].

The model was trained using the training set and validated on the validation set to monitor performance. After training, the model was evaluated on the test set, and the results were compared with those of the two baseline methods.

## 5. Results and Discussion

**Figure 6:**
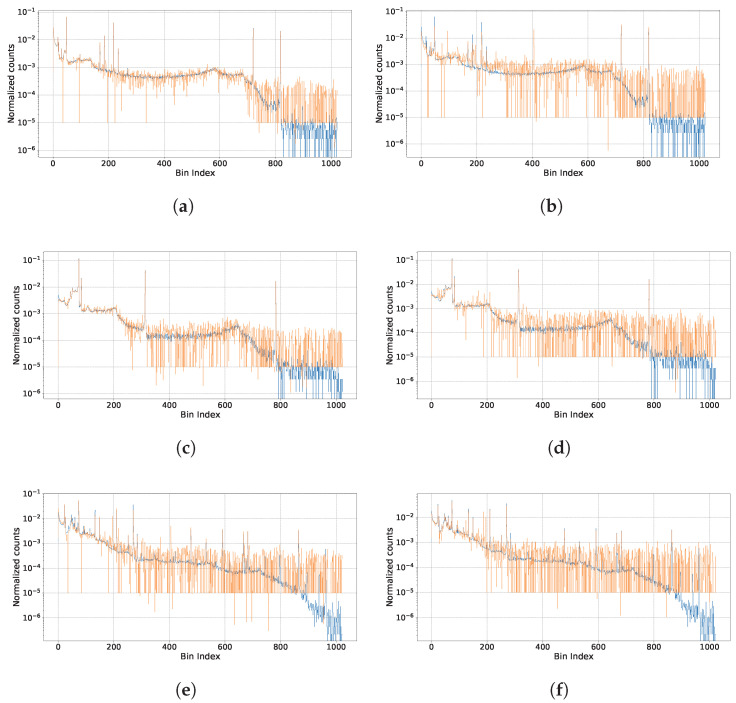
Pile-up spectrum recovery results of our methods, under different high count rates and multiple sources measurements. (**a**) λ=0.055, DC=1.12, P=0.674, Co60,Ba133, KL = 0.07, MSE = $0.7\times 10^{-7}$. (**b**) λ=0.265, DC=5.41, P=0.996, Co60,Ba133, KL = 0.35, MSE = $32.14\times 10^{-7}$. (**c**) λ=0.09, DC=1.84, P=0.841, Co57,Na22, KL = 0.06, MSE = $0.80\times 10^{-7}$. (**d**) λ=0.23, DC=4.69, P=0.991, Co57,Na22, KL = 0.17, MSE = $3.37\times 10^{-7}$. (**e**) λ=0.195, DC=3.98, P=0.981, Eu152,Ac225, KL = 0.20, MSE = $9.02\times 10^{-7}$. (**f**) λ=0.265, DC=5.41, P=0.996, Eu152,Ac225, KL = 0.24, MSE = $7.43\times 10^{-7}$. The results demonstrate that our method achieves highly accurate peak estimation for increasing levels of pile-up and spectral distortion. The blue curve represents the real energy spectrum, and the orange curve represents the estimated result of our proposed method.

The proposed method shows significant improvement in the energy spectrum recovery under high-count-rate conditions when compared to conventional techniques. As shown in Table 3 and Figure 6, the KL divergence and MSE values for our method are consistently lower than those for the baseline methods across all count rate levels. Also, our method achieves an accurate estimation effect when calculating the ER and FWHM metric, as shown in Table 4.

### 5.1. Visualization

The visualized results are shown in Figure 6, where the blue curve represents the ground truth spectrum (labels), and the orange curve denotes the estimated spectrum.

The experiment was conducted under different signal pile-up levels, quantified by λ, and varying distortion complexities (DC). The values of λ range from 0.055 to 0.265, while DC ranges from 1.12 to 5.41, and pile-up probability(P) from 0.674 to 0.996. These parameters simulate increasing levels of pile-up and spectral distortion.

The results demonstrate that our method achieves highly accurate peak estimation across all conditions:

- In low pile-up conditions (e.g., Figure 6a, λ=0.055, DC=1.12, P=0.674), the estimated peaks closely align with the ground truth spectrum, showcasing minimal deviations.
- As the pile-up level increases (e.g., Figure 6f, λ=0.265, DC=5.41, P=0.996), slight deviations are observed, particularly in higher-energy regions. Nevertheless, the primary peaks remain well-estimated, and the overall spectral trends are preserved.
- Across all cases, the proposed method effectively reconstructs the peaks, even under severe pile-up conditions, highlighting its robustness and accuracy.

The experimental results validate the effectiveness of our method in accurately estimating spectral peaks under high-count rate scenarios. Despite increasing levels of pile-up and distortion, the method maintains reliable performance, demonstrating its robustness for applications in high-activity environments.

### 5.2. Quantification

In Table 3, we compare KL divergence and MSE metric of energy spectrum estimations using our proposed method against two conventional algorithms: one without pulse correction (Method 1) and the fast correction algorithm (Method 2) [21]. These comparisons are made using different count rates and various sources. In both multi-source and single-source scenarios, the KL divergence and MSE are significantly lower compare to Method 1 and Method 2, indicating more accurate spectrum estimation with smaller errors.

At a high activity (λ=0.055), Method 3 shows the best performance (KL∼0.05, MSE∼0.27), while Methods 1 and 2 perform poorly (KL > 2.5, MSE > 90). When activity reaches (λ=0.095, 0.135), Method 3 remains robust, while Methods 1 and 2 worsen. Finally, when the pile-up effect becomes more severe (λ=0.175), Method 3 still performs well, while traditional methods have high KL (>5) and MSE (>900), highlighting its ability to recover the energy spectrum even in ultra high-count scenarios.

The results show that the model performs well across a range of activities, demonstrating its robustness and ability to generalize to unseen data. A critical challenge for traditional algorithms is their performance degradation as the count rate increases. Our approach incorporates count rate information as a conditioning factor, allowing it to maintain stable and accurate performance even under extremely high count rates. This robustness ensures reliable energy spectrum estimation, where traditional methods would typically fail or yield significant errors.

To better demonstrate the estimation effect of our model on the energy spectrum in more detail, we selected three sources to conduct experiments at different count rates to evaluate the model’s prediction results for the peak value. Specifically, the ER metric and the Full Width at Half Maximum (FWHM) are evaluated and shown in Table 4. It can be seen that our model can accurately estimate both the peaks in the low-energy region and the peaks in the high-energy region, and the ER in the low-energy region is slightly better than that in the high-energy region. When the count rate gradually increases, the ER increases; however, until the pile-up probability increases to 0.996, the estimation error of our method for the high-energy peak increases significantly. Under high counting rate conditions, the model can effectively suppress the pile-up effect, and the peak width of the estimated energy spectrum is in a very small range (with FWHM < 3 bins), which shows that our method has effective and stable pile-up correction capability in high activity environments.

In order to explore the impact of Energy–Duration matrix inputs of different dimensions on model performance and the accuracy of estimation results, we conducted experiments using matrices of four different sizes, as shown in Table 5. Note that since the input dimension of [256×4096] is large, the training batch size is unified to 4 to ensure the consistency of training. The data show that in general, the input dimension of [64×2048] will obtain the best results while the input dimension of [128×2048] will obtain the worst results. The reason why the effect deteriorates when the duration value increases to 128 but the energy remains unchanged at 2048 is that the matrix becomes sparse, that is, it does not contain more information about the stacking clusters. For example, when a stacking cluster of length 128 appears, its energy value is likely to exceed 2048, so it is not recorded. This also explains why when the input dimension increases to [128×4096], the estimation effect is better than that of [128×2048]. We did not show the result of [64×4096] because the probability of the element appearing in high-energy photons is extremely low, the stacking clusters with a length of 64 must all be high-energy particle events, and the main peaks of the elements also appear in the low-energy region.

A smaller matrix size can avoid the problem of information sparsity, which means that the model can capture more relevant features without being disturbed by redundant information, while larger matrix sizes may lead to over-fitting, especially when there is insufficient training data. Smaller matrices force the model to focus on more essential features by limiting the input dimension, which helps improve its generalization ability on the validation set. Furthermore, a larger matrix will require many more samples to provide a histogram that is statistically relevant, challenging the signal preprocessing. Increasing the input matrix dimension will sharply increase the model training cost with more training time and occupy larger memory of GPUs; however, it gives worse results. Overall, [64×2048] is proved to be the optimal training input size in our experiments.

**Figure 7:**
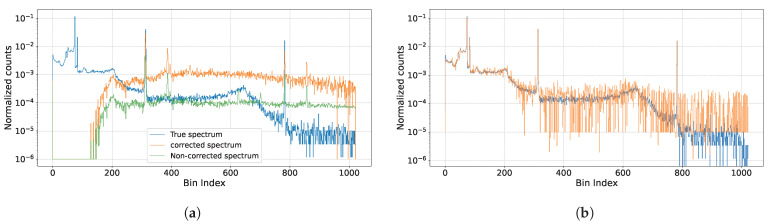
Comparison of spectra estimated by different traditional methods and our DL method shows that our approach provides more accurate estimations. The provided conditions are of λ=0.095, DC=1.94, P=0.856, Co57,Na22. (**a**) The blue curve is the true spectrum, the green curve is Method 1’s result, and the orange curve represents Method 2’s result. (**b**) The blue curve is the true spectrum, the orange curve is our proposed method’s result.

To better illustrate the pile-up spectrum recovery performance of different methods, we plot the predicted spectrum of different methods in Figure 7, where Figure 7a shows the spectrum estimated by the traditional methods, and Figure 7b shows the predicted spectrum by our method. The result shows that the spectrum predicted by the pile-up correction algorithm has a smaller peak error; however, it fails at estimating low-energy region area. Our proposed DL algorithm, focuses on these two flaws, both low-energy peaks and high-energy peaks have less prediction error.

## 6. Conclusions

In this study, we proposed a DL-based algorithm for pile-up correction in spectrometric data under high-count-rate measurements. Our approach leverages the power of a 2D-UNet architecture, combined with a count rate embedding module, utilizing both spatial and temporal features to effectively recover the energy spectrum distorted by pile-up effects. The method utilizes attention gates to focus on the relevant portions of the input distorted Energy–Duration matrix, significantly improving the accuracy of energy recovery, even under challenging high-count-rate conditions.

Through comprehensive experimentation, we demonstrated that our method outperforms traditional pile-up correction techniques in both low and high-count-rate scenarios. Specifically, our model consistently showed lower KL divergence and MSE, indicating superior recovery of the energy spectrum.

Furthermore, our model exhibits strong robustness and generalization performance across varying unseen count rate levels, with minimal performance degradation as the pile-up degree increases. This indicates the adaptability of our method to different measurement conditions, making it a reliable tool for spectrometric applications in fields such as nuclear physics, environmental monitoring, and medical diagnostics.

The proposed deep learning-based pile-up correction method offers a novel and effective solution to the challenges posed by high-count-rate measurements. It significantly improves the accuracy and reliability of energy spectrum recovery, with strong potential for further enhancement and deployment in real-world spectrometric systems. In future work, we plan to extend this work by integrating a pre-trained count rate estimator neural network into the model, which can predict the count rate directly from raw signals, eliminating the reliance on known count rate information. To further address the real-world detector’s nonlinear response as well as the dead time effect, a more comprehensive approach is needed to account for the challenges; our future work will extend the current model as well as refine the choice of simulation parameters to incorporate these factors.

## Figures and Tables

**Table 1:** Simulator parameter settings during data generation.

Parameter	Value
Count rate (λ)	0.05~0.3
Energy bins (*B*)	1024
Noise level (σ)	$10^{-1}$
Signal length per sample (L)	0.2048
Sampling rate ($f_{S}$)	$10^{7}$
Shape type	double exponential
Shape factor	$\tau_{1}$: $\mu =3*10^{-7},\sigma =10^{-8}$ $\tau_{2}$: $\mu =10^{-8},\sigma =10^{-10}$

**Table 2:** Experimental settings.

Training Parameters	Value
Batch size	16
Epoch	200
Learning rate	0.0001
Optimizer	Adam
Train sample	1440
Test sample	360
Model depth	5
Hidden size	64
Kernel size	2
Stride	2

**Table 3:** The table shows the KL and MSE score calculated by three algorithms under different λ, from 0.055 to 0.295, P from 0.674 to 0.996. Multi-sources are mixed with a weight of 1:1, and the symbol ‘-’ indicates that the algorithm fails under the current conditions. The results show that the proposed method outperforms both Method 1 and Method 2.

		Multi-Source												Single-Source							
Activity	Source Name	Co60,Ba133		Cs137,Co57		Ba133,Eu152		Co57,Na22		Eu152,Ac225		Na22,Am241		Cr51		Er169		^197*m*^Hg		I125	
$\lambda_{n}$	**Metrics(/1 × $10^{-5}$)**	KL	MSE	KL	MSE	KL	MSE	KL	MSE	KL	MSE	KL	MSE	KL	MSE	KL	MSE	KL	MSE	KL	MSE
0.055	Method 1	2.69	96.03	3.20	330.44	2.76	147.35	2.54	295.69	2.82	91.95	2.29	730.43	3.40	885.63	2.75	223.97	3.39	201.97	0.50	419.23
	Method 2	2.97	89.68	2.98	307.29	2.68	144.24	2.52	285.18	2.79	90.98	2.19	713.27	3.51	892.72	2.45	218.25	3.43	182.00	0.28	352.35
	Method 3 (Ours)	**0.07**	**0.70**	**0.08**	**0.44**	**0.07**	**0.46**	**0.08**	**0.86**	**0.06**	**0.68**	**0.09**	**4.80**	**0.05**	**0.27**	**0.07**	**19.44**	**0.08**	**0.40**	**0.08**	**0.60**
0.095	Method 1	3.05	101.82	4.35	347.86	3.35	156.91	3.55	305.31	3.32	97.89	3.62	748.12	3.20	939.75	3.97	253.52	3.87	222.89	1.48	663.49
	Method 2	4.10	96.47	5.25	329.31	3.77	158.07	4.30	294.98	3.89	98.04	4.19	739.54	3.81	850.03	4.13	260.33	4.76	187.65	1.14	615.32
	Method 3 (Ours)	**0.06**	**213.81**	**0.07**	**0.40**	**0.09**	**1.25**	**0.06**	**0.63**	**0.11**	**1.48**	**0.06**	**0.45**	**0.08**	**2.96**	**0.07**	**15.03**	**0.09**	**0.38**	**0.04**	**0.11**
0.135	Method 1	3.38	103.04	4.57	352.06	3.44	158.91	3.45	309.62	3.32	99.82	3.62	751.75	2.72	968.47	4.37	259.26	4.17	236.14	3.10	774.69
	Method 2	4.79	95.18	6.21	326.73	4.83	157.68	5.07	297.46	4.97	99.84	5.14	740.70	3.98	884.49	6.03	288.89	5.89	233.80	4.301	931.57
	Method 3 (Ours)	**0.06**	**0.74**	**0.07**	**0.49**	**0.07**	**1.26**	**0.11**	**1.27**	**0.12**	**1.43**	**0.11**	**0.75**	**0.260**	**72.87**	**0.09**	**0.64**	**0.1**	**0.40**	**0.04**	**0.07**
0.175	Method 1	3.30	103.82	4.25	354.77	3.28	159.87	3.65	311.28	3.16	100.40	3.51	753.43	2.86	978.56	4.21	260.36	3.90	236.81	3.91	808.39
	Method 2	5.20	99.69	6.41	321.99	5.60	163.61	5.76	311.84	5.53	102.83	5.57	752.71	4.59	974.64	7.29	408.54	6.19	231.41	9.40	-
	Method 3 (Ours)	**0.09**	**1.21**	**0.10**	**0.88**	**0.22**	**31.72**	**0.14**	**3.84**	**0.12**	**3.14**	**0.08**	**0.47**	**0.24**	**11.64**	**0.10**	**0.50**	**0.23**	**2.03**	**0.06**	**0.15**
0.215	Method 1	3.01	104.30	3.65	355.84	3.08	159.90	3.64	311.34	2.86	100.41	3.45	753.55	3.17	978.93	3.83	260.69	3.42	237.40	4.04	822.34
	Method 2	5.46	105.58	6.30	321.11	6.00	161.84	6.20	312.13	5.85	101.50	6.20	753.70	5.95	980.13	8.260	117.78	6.27	233.68	11.77	-
	Method 3 (Ours)	**0.12**	**1.18**	**0.12**	**0.58**	**0.32**	**46.21**	**0.22**	**9.80**	**0.22**	**4.74**	**0.22**	**7.55**	**0.53**	**124.44**	**0.16**	**1.90**	**0.37**	**7.94**	**0.05**	**0.09**
0.255	Method 1	2.47	104.50	2.94	356.43	2.95	159.90	3.38	311.37	2.59	100.40	3.25	753.56	2.83	979.03	3.42	260.86	3.35	237.53	3.86	826.66
	Method 2	5.69	101.06	6.46	333.69	6.22	161.14	6.47	311.65	6.21	101.33	6.48	753.36	6.35	980.00	8.97	5021.00	6.97	239.46	12.65	-
	Method 3 (Ours)	**0.35**	**8.02**	**0.25**	**5.01**	**0.44**	**63.32**	**0.24**	**4.62**	**0.59**	**47.49**	**0.28**	**12.11**	**0.77**	**529.59**	**0.16**	**1.76**	**0.62**	**25.68**	**0.05**	**0.19**
0.295	Method 1	2.09	104.61	3.77	356.58	6.74	159.90	3.92	311.41	2.09	100.45	2.53	753.60	2.52	979.02	3.40	260.87	4.30	237.54	3.80	828.18
	Method 2	6.68	107.30	6.61	340.03	2.88	161.51	6.67	311.98	6.66	101.52	6.84	753.53	6.59	980.29	7.59	291.10	7.48	239.89	13.20	-
	Method 3 (Ours)	**0.86**	**107.95**	**0.60**	**30.45**	**0.51**	**43.16**	**0.23**	**5.49**	**0.66**	**67.48**	**0.30**	**6.01**	**0.96**	**419.36**	**0.35**	**22.53**	**0.76**	**58.33**	**0.08**	**0.71**

The MSE metric is in units of $10^{-7}$.

**Table 4:** The table shows the ER and FWHM metrics of the energy spectrum estimated by the model, calculated from the peak values of the sources. The experiments were carried out under different count rates under different λ, from 0.055 to 0.295, P from 0.674 to 0.996 and different sources: Co60,Cs137, and Am241. The results show that our model can accurately estimate the peak value at high count rates in most cases.

	Single Sources									
	Co60				Cs137				Am241	
Activity	1173.24		1332.50		31.80		661.66		59.54	
$\lambda_{n}$	ER	FWHM	ER	FWHM	ER	FWHM	ER	FWHM	ER	FWHM
0.055	0.073	2.521	0.076	2.520	0.004	0.805	0.032	2.620	0.042	2.503
0.105	0.019	2.520	0.016	2.516	0.009	2.802	0.002	2.620	0.014	2.503
0.135	0.014	2.522	0.013	2.515	0.028	0.818	0.029	2.620	0.004	2.503
0.175	0.023	2.520	0.018	2.518	0.023	2.836	0.040	2.620	0.004	2.503
0.245	0.159	2.519	0.163	2.529	0.046	0.818	0.099	2.623	0.001	2.504
0.295	0.587	2.519	0.521	2.568	0.158	1.698	0.355	2.620	0.077	2.504

The peak position and FWHM are represented by energy (keV) and bins, respectively. One bin represents 1.6 keV.

**Table 5:** The model estimation results were obtained by using the Energy–Duration matrix of different scales as the input training data of the neural network, using KL and MSE as evaluation indicators. The experiments were carried out under different count rates from 0.055 to 0.255, P from 0.674 to 0.994, and different sources: Cr51,Er169, and I125. The smallest Energy–Duration matrix has the best performance.

		Single Sources					
Energy–Duration	Activity	Cr51		Er169		I125	
	$\lambda_{n}$	KL	MSE	KL	MSE	KL	MSE
2048×64	0.055	0.03	0.22	0.03	0.18	0.02	0.03
	0.135	0.14	34.18	0.04	0.50	0.02	0.11
	0.255	0.50	243.71	0.18	9.42	0.03	0.11
2048×128	0.055	0.11	1.47	0.08	1.08	0.08	7.97
	0.135	0.07	0.74	0.08	0.55	0.06	2.13
	0.255	0.81	607.47	0.26	11.52	0.04	0.26
4096×128	0.055	0.03	0.66	0.02	0.08	0.03	0.87
	0.135	0.04	1.71	0.04	0.53	0.02	0.10
	0.255	0.66	538.34	0.20	24.70	0.02	0.11
4096×256	0.055	0.07	0.80	0.03	0.04	0.05	0.13
	0.135	0.07	1.99	0.05	0.14	0.03	0.14
	0.255	0.80	604.16	0.23	14.45	0.03	0.16

The MSE metric is in units of $10^{-7}$.

## Data Availability

The data presented in this study are available on request from the corresponding author. The data are restricted due to ongoing intellectual property development.
